# Ultraviolet-Visible Spectroscopy and Chemometric Strategy Enable the Classification and Detection of Expired Antimalarial Herbal Medicinal Product in Ghana

**DOI:** 10.1155/2021/5592217

**Published:** 2021-06-24

**Authors:** Jacob N. Mensah, Abena A. Brobbey, John N. Addotey, Isaac Ayensu, Samuel Asare-Nkansah, Kwabena F. M. Opuni, Lawrence A. Adutwum

**Affiliations:** ^1^Department of Pharmaceutical Chemistry, Faculty of Pharmacy and Pharmaceutical Sciences, College of Health Sciences, KNUST, Kumasi, Ghana; ^2^Department of Pharmaceutical Chemistry, School of Pharmacy, College of Health Sciences, University of Ghana, Accra, Ghana

## Abstract

To meet the growing demand for complementary and alternative treatment for malaria, manufacturers produce several antimalarial herbal medicinal products. Herbal medicinal products regulation is difficult due to their complex chemical nature, requiring cumbersome, expensive, and time-consuming methods of analysis. The aim of this study was to develop a simple spectroscopic method together with a chemometric model for the classification and the identification of expired liquid antimalarial herbal medicinal products. Principal component analysis model was successfully used to distinguish between different herbal medicinal products and identify expired products. Principal component analysis showed a clear class separation between all five herbal medicinal products (HMP) studied, with explained variance for first and second principal components as 37.51% and 26.38%, respectively, while the third principal component had 18.74%. Support vector machine classification gave specificity and accuracy of 1.00 (100%) for training set data for all the products. The validation set HMP1, HMP2, and HMP3 had sensitivity, specificity, and accuracy of 1.00. HMP4 and HMP5 had sensitivity and specificity of 0.90 and 1.00, respectively, and an accuracy of 0.98. The support vector machine classification and principal component analysis models were successfully used to identify expired herbal medicinal products. This strategy can be used for rapid field detection of expired liquid antimalarial herbal medicinal products.

## 1. Introduction

The worldwide mortality attributed to malaria in 2019 was 409,000 out of which 384,000 occurred in Africa, with most of them being children and pregnant women [1]. These mortality cases in Africa represent over 93% of all malaria-related deaths worldwide. Of the 87 malaria endemic regions in the world, 28 African countries and India accounted for 95% of malaria cases reported globally [1]. Over the years, concerns have risen about the effectiveness and safety of orthodox antimalarial drugs, coupled with the development of resistance to drugs used in the treatment of malaria [2]. Artemisinin combination therapy was introduced to reduce the rate of development of resistance. However, the associated side effects and adverse drug reactions have made it unattractive for some patients [3], as well as cost of treatment, treatment failures, and accessibility.

In such situations, plant medicines have provided a viable alternative to orthodox medicines for the treatment of malaria. In Ghana, plant medicines remain a major source of antimalaria therapy, as a large number of Ghanaians are observed to patronize herbal antimalaria remedies. Such preference stems from the lower costs of products, perceived better efficacy and reduced side effects, and acceptability based on peer recommendation. Treatment outcomes from the use of these herbal remedies have been positive in a number of cases because medicinal plants have been sources of many bioactive compounds including natural scaffolds for antimalarial drugs such as quinine and artemisinin [4, 5]. These plant medicines may circumvent the challenges of parasite resistance and toxicity by the synergistic activity of the several constituent secondary metabolites [6].

Plant medicines are normally formulated as herbal medicinal products (HMPs) with optimum pH and minimal toxicity due to internal buffering effect [5] and relatively low concentration of constituent phytochemicals, respectively. Currently, the prevalence of use of HMPs in Ghana is about 76% [7]. Considering the debilitating nature of malaria and the potential for development of organ and neurological complications in severe cases, there is the need for surveillance and continuous quality monitoring of HMPs to safeguard the integrity of the products in order to safeguard the general public. However, the efficient quality monitoring of HMPs still remains a major challenge. In the case of orthodox medicines, an assay can be performed to determine the levels of active ingredients and other impurities that may be present. On the other hand, HMPs usually have several phytochemical constituents that nearly make it impossible to identify all the bioactive compounds and accordingly have them quantified, more especially when the products are polyherbal. In spite of this, variations in the levels of phytoconstituents in plant materials collected from different sources and at different times are a major concern for the monitoring of HMPs for content uniformity, especially in situations where the products are used for the treatment of infectious diseases such as malaria. It is already known that the levels of plant secondary metabolites are influenced by factors such as growth conditions, time and method of harvesting, and storage condition, as well as the geographical location [8–11]. Since at the moment a lot of these factors influence the levels of phytoconstituents in medicinal plants, and the manufacturing practices are not standardized, it becomes important in the interest of the HMP patrons to develop simple, cost-effective, and efficient system to assure the quality of HMPs.

In view of the difficulty associated with the development of assay methods for HMPs because of the myriad of secondary metabolites, the approach by the Food and Drugs Authority, Ghana, to approve and register HMPs has mainly focused on microbial load, toxicity, pH, and some physicochemical parameters such as ash value and acid value. It is however necessary to have a technique where the protocol involves measurement of some phytochemical components of the HMPs. Some of the challenges associated with such desired methods in resource constrained environments are the cost of equipment, accessories, and maintenance. In addition, the methods would be expected to target detection and quantification of almost all the phytochemical components of the polyherbal HMPs or selected markers [12]. These methods usually involve techniques such as high-performance liquid chromatography (HPLC), gas chromatography-mass spectrometry (GC-MS), and capillary electrophoresis (CE) which are difficult to find in countries such as Ghana for routine chemical quality monitoring of HMPs. Besides the instrumental challenges are tedious sample preparations with high volume of solvent and solvent-wasting separation methods [12, 13].

The ultraviolet-visible (UV-Vis) spectroscopy as a simple, cost-effective, and nondestructive technique has found applications in environmental, pharmaceutical, and other related fields. For example, the British and United States Pharmacopoeias employ UV-Vis-based methods for the assay of some pharmaceutical products, as well as adjunct method for the identification of certain active pharmaceutical ingredients. The technique has also been reported to be useful for the analysis of liquid HMPs [14], and the inherent advantages are that the methods are easy to use, enabling laboratories to adopt and effectively implement the protocols without any extensive training of technical staff. In UV-Vis spectroscopy, it has been observed that spectra obtained from complex mixtures such as liquid HMPs are usually highly convoluted. This is because the absorption spectra of several components within the complex samples are superimposed on each other. The authors, however, envisaged that the application of chemometric techniques to the complex UV-Vis data can lead to useful analyses with relevant conclusions on the quality of HMPs.

Chemometric methods involve the use of mathematical and statistical tools to extract useful information from complex data [15]. These methods have been around for a while but have become popular lately due to the availability of easy-to-use software and statistical packages [16, 17]. Chemometric methods are generally used for machine learning and optimization of experiments (i.e., design of experiment) [18, 19]. This work will focus on the former as the latter is beyond the scope of this paper. Machine learning methods are usually termed as supervised or unsupervised [20]. Unsupervised learning methods such as principal component analysis (PCA) are arguably the most used chemometric method [20, 21]. PCA is a dimensionality reduction technique which reveals inherently hidden patterns in data. In a PCA model, samples of the same class, which have similar attributes, are clustered closer to each other. Thus, a confidence ellipse generated around a cluster of samples would show high variability in the score space. Samples deviating from the standard product will be seen projecting further away from the center of that cluster. In the context of our study, this can provide guidance in the detection of unwholesome or expired HMPs. Supervised learning methods, such as support vector machines (SVM), can also be used to classify liquid HMPs in order to distinguish them from other products. Thus, two different antimalarial HMPs are expected to be classified into two different groups. In addition, variations in the chemical composition due to expiration of the product can also be determined as the UV-Vis chemical fingerprint of the product becomes altered. Such alteration, detectible with PCA or SVM, can be employed to identify expired HMPs.

Therefore, in this study, we propose a routine for the classification of liquid antimalarial HMPs using SVM and UV-Vis spectroscopy. We further demonstrate the use of PCA and SVM models to monitor the variations in liquid HMPs and to identify expired products. This approach has the potential to be expanded to the detection of adulterants in liquid antimalarial HMPs and by extension, other HMPs.

## 2. Experimental

### 2.1. Sample Preparation

Five liquid antimalarial HMPs were obtained from pharmacies and herbal shops in Accra and Kumasi, Ghana. The antimalarials used for this study were packaged in 500 mL amber colored plastic bottles. To ensure anonymity, the samples were coded HMP1, HMP2, HMP3, HPM4, and HMP5. Three different batches each were obtained for each sample. A fourth batch consisting of expired HMP4 labelled HMP4X was also obtained from a herbal shop.

A 5 mL portion of each sample was pipetted into a 50 mL volumetric flask. Distilled water was added to the volumetric flask to make up to the 50 mL mark. This yielded 5% (v/v) concentration of the liquid herbal antimalarial in distilled water.

### 2.2. Data Collection

UV-Vis spectra were collected using a JENWAY 7315 UV-Vis spectrophotometer (Jenway, UK) equipped with a Perkin Elmer Spectrum (Spectrum Two, Version 10.03.09, Serial Number 94133, Waltham, USA). The samples were analyzed using a 1 mL fused silica cuvette and at a wavelength range of 200 to 700 nm using distilled water as blank/reference. At least ten repeated scans were made for each sample. The raw data from the spectrum was imported into MATLAB R2020b (The MathWorks®, Natick, MA, USA). PCA and SVM models were generated using PLS Toolbox 8.9 (Eigenvector Research Inc., Manson, WA, USA).

### 2.3. Data Processing and Analysis

The data was organized into a matrix of samples in rows and wavelength in columns. The dataset matrix consisted of 167 × 501 (samples × wavelengths). Spectral data was smoothed with a moving average filter using a window of five. The spectral data was subsequently decluttered using generalized least squares- (GLS-) based weighting strategy using an alpha value of 0.02 [22]. Each column of the data matrix was mean centered while the rows were normalized to 1.

The data was further split into two main groups: 2/3 for model training and optimization and a third for external validation sets. The training and validation set data consisted of 111 and 56 samples, respectively.

Discriminant variable (DIVA) test was performed on the training set data to identify regions of the spectra providing information about the important regions of the data.

The PCA model was generated with the training and optimizing set data. In the PCA model, the variance explained by the model by each component is represented as a percentage. It is used to demonstrate measure of discrepancy between the model and the data. Thus, a higher explained variance is desired. The validation data were subsequently projected into the PCA model.

The training data was used to generate SVM classification models for the 5 classes of samples. The validation data was then projected into the model to evaluate the performance on an external validation set. The evaluation was based on the model's sensitivity, specificity, and accuracy [23]. A model's ability to predict positive samples is true positive rate/sensitivity (sensitivity = true positives (TP)/number of positives (NP)). Specificity measures the model's ability to correctly identify negative samples, also known as true negative rate (specificity = true negatives (TN)/number of negatives (NN)). Accuracy measures the overall true predicting power (accuracy = (TP + TN)/(NP + NN)). These metrics are scaled from zero to 1, with 0 and 1 being the worst and best model, respectively.

The SVM model was further tested on the HP4X, which are the expired HP4 samples. PCA models were also generated and evaluated with the training and validation sets, respectively.

## 3. Results and Discussion

The assessment of quality of HMPs in Ghana has largely been based on some organoleptic and physicochemical parameters including but not limited to color, pH, and microbial load. However, it is necessary to develop advanced but simple strategies that target the phytoconstituents of HMPs in the evaluation of chemical quality. It is known that variation in these secondary metabolites supposed to be responsible for the biological activities of HMPs exists as a result of factors including environment, harvesting, manufacturing, storage, and stability. Due to the presence of myriad of chemical compounds in polyherbal products and the lack of adequate robust analytical methods to check batch-to-batch consistency in levels of phytoconstituents and identify expired or decomposed HMPs that may not show perceptible physical changes, unscrupulous persons could rebottle and sell substandard and unwholesome products to the general public. Therefore, this study has explored the application of UV-Vis spectroscopy and chemometric analysis in dealing with the problem. Antimalarial HMPs were chosen as a test case due to their high demand and over-the-counter usage.

**Figure 1:**
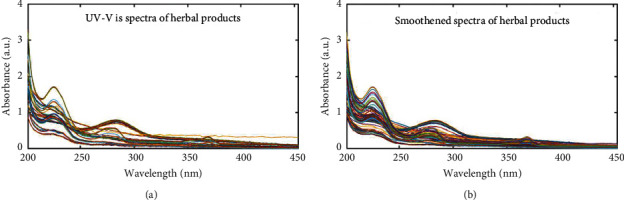
UV-Vis spectra of liquid herbal medicinal products. (a) Raw data. (b) Spectra data smoothed with a moving average filter using a window of 5.

Generally, all the UV-Vis spectra obtained from the analyses of the liquid antimalarial HMPs showed maximum absorbance around 230 nm, 280 nm, and 375 nm (Figure 1(a)), which suggests the presence of compounds with conjugated systems or chromophores and the suitability of our choice of technique. This agrees with other findings that show the presence of chromophoric compounds in plant products [12].

**Figure 2:**
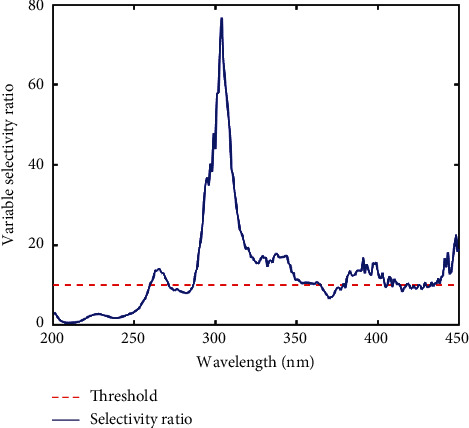
Selectivity ratio plot of UV-Vis spectra of liquid HMP obtained from DIVA test showing feature importance to discrimination between classes (*y*-axis) and wavelength (*x*-axis). Wavelengths, at which the SR is less than the threshold (red line), were eliminated from the data.

Due to noise in the dataset, a moving window smoothing algorithm was implemented (Figure 1(b)), which showed similar spectra characteristics relative to the raw spectra. In order to identify the more informative regions of the spectra, discriminant variable analysis was performed as previously described [24] to generate a variable selectivity ratio (SR) plot (Figure 2). In this analysis, an SR value less than 10 was considered as one with low discrimination power and, thus, was eliminated from the data. The threshold shows the variables which were above the set limit and reduced the number of variables from 501 to 324. It must be emphasized that a fewer number of descriptors with better discriminating power are much more desirable as they lead to simpler models [25, 26].

**Figure 3:**
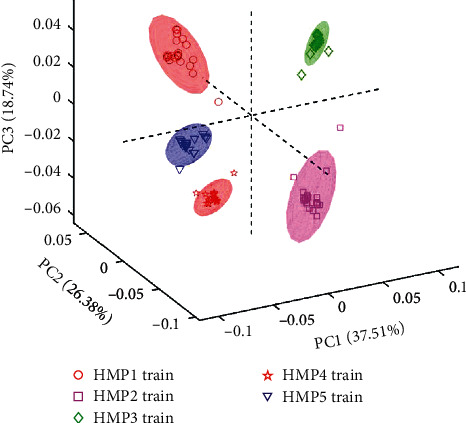
PCA score plot for training set data. A score plot for PC1 (37.51%) vs. PC2 (26.38%) vs. PC3 (18.74%). HMP1—red circles, HMP2—purple squares, HMP3—green diamonds, HMP4—red pentagrams, and HMP5—blue triangles. Training and validation data are represented by hollow and filled markers, respectively.

**Figure 4:**
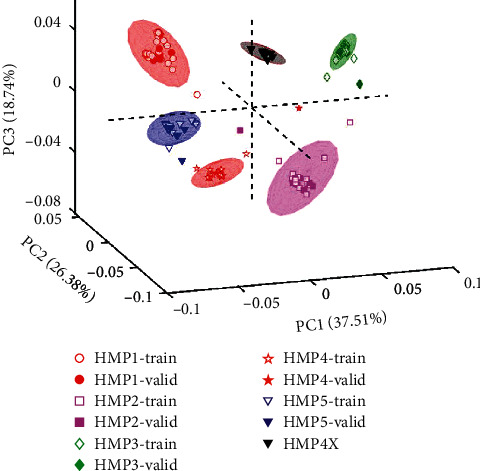
PCA plot for PC1 (37.51%) vs. PC2 (26.38%) vs. PC3 (18.74%) for training set, validation set, and expired products HMP4X. HMP1—red circles, HMP2—purple squares, HMP3—green diamonds, HMP4—red pentagrams, and HMP5—blue triangles. Black triangles: HMP4X. Training and validation data are represented by hollow and filled markers, respectively.

Next, principal component analysis was performed using the training set data (only the 324 variables). The results show a PCA score plot of PC1 vs. PC2 vs. PC3 (Figure 3). The explained variances for PC1 and PC2 were 37.51% and 26.38%, respectively, while the third principal component had 18.74%. Thus, a total explained variance of 82.63% was observed. It can be seen in this three-dimensional score space that the various antimalarial HMPs are all clustered in separate groups. A 95% confidence ellipse generated around each cluster shows quite a few of the training set samples straying out of the cluster. This demonstrates that UV-Vis spectra and PCA models can be used to distinguish various liquid antimalarial HMPs. Subsequently, the external validation set of each HMP class was projected in the model (Figure 4, where HMP1 are red circles, HMP2 are purple squares, HMP3 are green diamonds, HMP4 are red pentagrams, and HMP5 are blue triangles). In Figure 4, samples used for the training and validation sets are represented by hollow and filled markers, respectively. Here it is also apparent that those samples that were not used in training the model are also projected into the correct subgroups.

The ability of the PCA model generated to identify expired products was also evaluated. Expired products from HMP4, labelled as HMP4X, were also projected into the model as shown in Figure 4. These expired products were clustered into a different score space (represented as black triangles). This further demonstrates that, using the UV-Vis spectra and PCA, products that are expired can be easily detected. This is of high importance due to the fact that liquid HMPs could go bad without showing perceptible variations in their physical appearance and taste.

**Figure 5:**
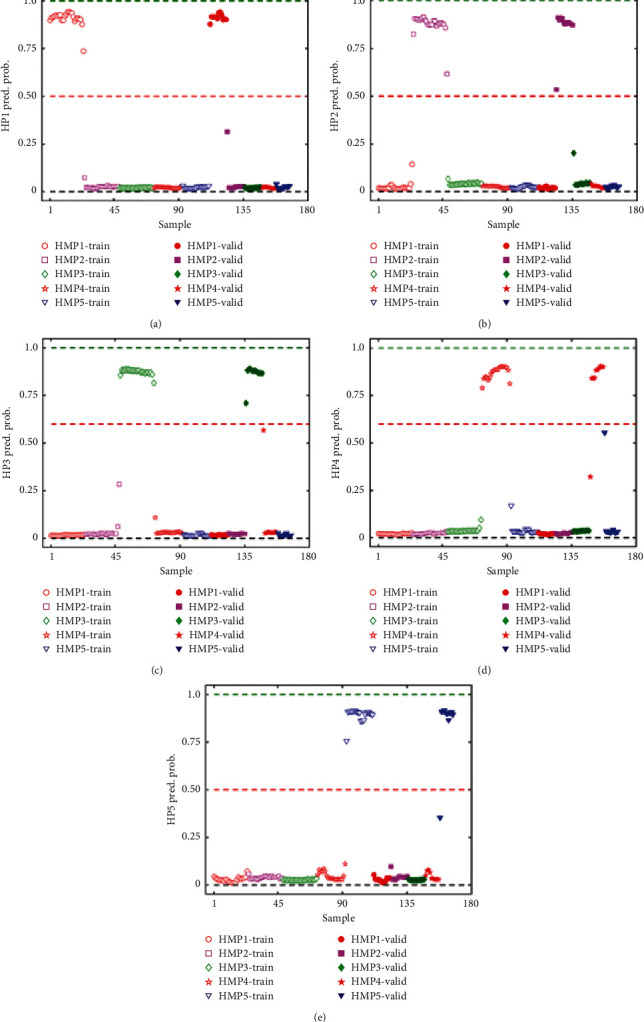
SVM class predicted probability for herbal medicinal products HMP1 (a), HMP2 (b), HMP3 (c), HMP4 (d), and HMP5 (e). HMP1—red circles, HMP2—purple squares, HMP3—green diamonds, HMP4—red pentagrams, and HMP5—blue triangles. Hollow and filled markers represent training and validation sets, respectively. The red dashed line represents the discrimination barrier above which samples are positively predicted as designated by the *y*-axis label.

SVM classification models were generated using the 324 features obtained from the DIVA test. The model was generated using a radial basis function kernel with cost and gamma values of 100 and 0.1, respectively. A venetian blind cross validation was employed due to structure of the data and it gave consistent results. The model was generated with the training set data and validated using an external validation set. The class predicted probability plots for all the 5 products (HMP1, HMP2, HMP3, HMP4, and HMP5) are shown in Figures 5(a)–5(e), where HMP1 are red circles, HMP2 are purple squares, HMP3 are green diamonds, HMP4 are red pentagrams, and HMP5 are blue triangles, red dash lines are discrimination barrier, and training and validation sets are represented by hollow and filled markers, respectively. In the SVM of Figure 5, class predicted probability closer to zero (black dash lines) indicated less likelihood of samples belonging to the class being predicted. On the other hand, a class predicted probability close to 1.00 (green dash line) indicates that the sample may belong to the class being predicted. A class discrimination boundary is represented by a red dashed line. The numerical results from Figure 5 are shown in Table 1. There were no false negative or false positives leading to classification sensitivity, specificity, and accuracy of 1.00 (denoting 100%), which demonstrate the power of combination of UV-Vis spectrum and SVM for HMPs classification. However, in the validation set, two false positives were identified from HMP4 and HMP5. It is normal for a classification model to perform better on a training set data than a validation set.

**Figure 6:**
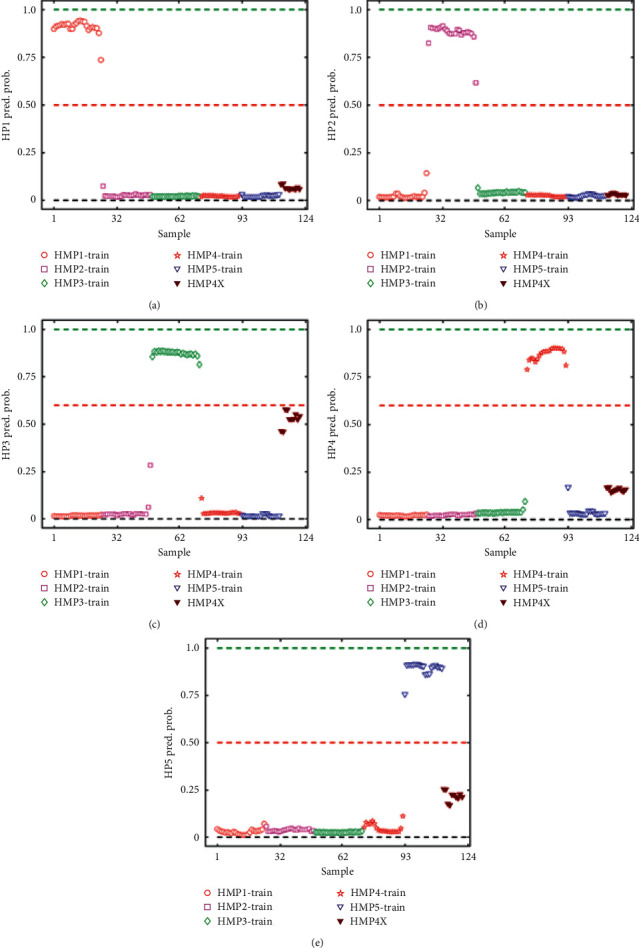
SVM class predicted probability for herbal medicinal products for training data HMP1 (a), HMP2 (b), HMP3 (c), HMP4 (d), and HMP5 (e) showing expired samples HMP4X. HMP1—red circles, HMP2—purple squares, HMP3—green diamonds, HMP4—red pentagrams, and HMP5—blue triangles. HMP4X—black triangles. Training and validation data are represented by hollow and filled markers, respectively. The red dashed line represents the discrimination barrier above which samples are positively predicted as designated by the *y*-axis label.

The ability of the SVM model to detect expired products was evaluated using the spectra of HMP4X. The samples in HMP4X were projected into the model to check, if indeed, the model will predict it as HMP4 or others. It can be seen that HMP4X samples are not predicted to be in any of the 5 HMP groups (Figure 6). Thus, the class predicted probability is below 0.5 for all five classes of HMPs. This is because these products have undergone some chemical changes even though there is no perceptible physical change which has altered their UV-VIS spectra. This demonstrates that, using UV-Vis spectra and SVM classification, expired herbal products can be detected. The study scope would be expanded to include batch-to-batch consistency and stability of HMPs.

## 4. Conclusion

A simple spectroscopic method (UV-Vis spectroscopy) together with chemometric models was successfully developed and applied to the assessment of liquid antimalarial HMPs. This method demonstrates the ability of PCA to distinguish between different HMPs. In addition, we applied SVM models to UV-VIS spectra of liquid HMPs to classify different antimalarials. Prediction sensitivity, specificity, and accuracy of 1.00 (100%) were observed for training set data for all the products. With respect to the validation set, sensitivity, specificity, and accuracy of prediction were 1.00 in all cases for HMP1, HMP2, and HMP3. However, sensitivity and accuracy for HMP4 and HMP5 were 0.90 and 0.98, respectively. The SVM method also demonstrated its ability to distinguish between wholesome and expired products.

## Figures and Tables

**Table 1:** Table of results for SVM classification for training and validation sets for all herbal products.

Product ID	True positive	False negative	True negative	False positive	Sensitivity	Specificity	Accuracy
*Training set*							
HMP1	24	0	87	0	1.00	1.00	1.00
HMP2	24	0	87	0	1.00	1.00	1.00
HMP3	24	0	87	0	1.00	1.00	1.00
HMP4	20	0	91	0	1.00	1.00	1.00
HMP5	19	0	92	0	1.00	1.00	1.00

*Validation set*							
HMP1	12	0	44	0	1.00	1.00	1.00
HMP2	12	0	44	0	1.00	1.00	1.00
HMP3	12	0	44	0	1.00	1.00	1.00
HMP4	9	0	46	1	0.90	1.00	0.98
HMP5	9	0	46	1	0.90	1.00	0.98

## Data Availability

The data for this project are available at lawrenceadutwum/herbalproducts (github.com).
